# Supplementary material for: TRPM7 is an essential regulator for volume-sensitive outwardly rectifying anion channel
Source: Commun Biol. 2021 May 20;4:599. doi: 10.1038/s42003-021-02127-9 (PMC8137958; doi:10.1038/s42003-021-02127-9)
Supplement: Supplementary file 2 — Supplementary Information [file 42003_2021_2127_MOESM2_ESM.pdf]

TRPM7 is an essential regulator for volume-sensitive outwardly rectifying anion channel

Tomohiro Numata[1], Kaori Sato-Numata[1,2], Meredith C. Hermosura[3], Yasuo Mori[4] & Yasunobu Okada[5,6,7]*

[1]Department of Physiology, Graduate School of Medical Sciences, Fukuoka University, Fukuoka 814-0180, Japan. [2]Japan Society for the Promotion of Science, Tokyo 102-0083, Japan. [3]John A. Burns School of Medicine, 651 Ilao St., Honolulu, HI 96813, USA. [4]Laboratory of Molecular Biology, Department of Synthetic Chemistry and Biological Chemistry, Graduate School of Engineering, Kyoto University, Kyoto 615-8510, Japan. [5]National Institute for Physiological Sciences, Okazaki 444-8787, Japan. [6]Department of Physiology, School of Medicine, Aichi Medical University, Nagakute 480-1195, Japan. [7]Department of Physiology, Kyoto Prefectural University of Medicine, Kyoto 602-8566, Japan.

Running title: TRPM7 is an essential regulator for VSOR

*Correspondence author. Yasunobu Okada MD, PhD. National Institute for Physiological Sciences, Okazaki 444-8787, Japan. Tel: +81-564-59-5262; Fax: +81-564-52-5263; E-mail: okada@nips.ac.jp

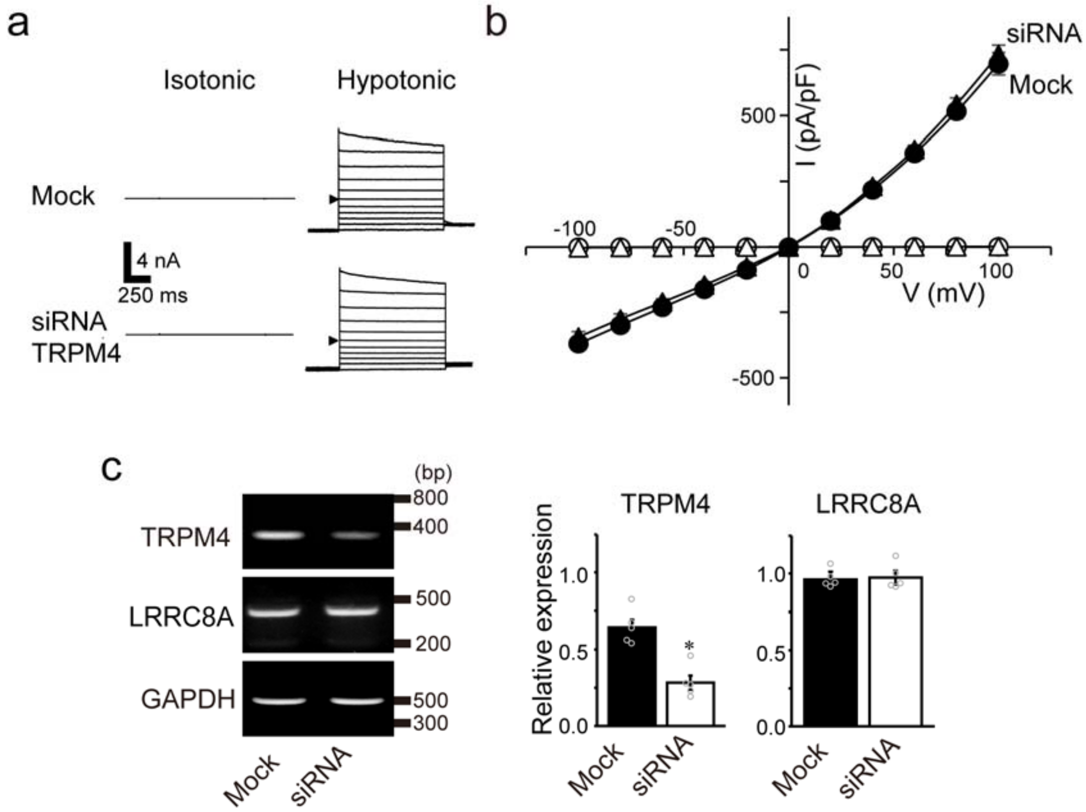

Supplementary Figure 1. TRPM4 expression is not involved in VSOR activity and LRRC8A expression in HeLa cells. a Representative whole-cell current traces elicited by step pulses in isotonic and hypotonic conditions in Mock-transfected (*Mock*) and siRNA-TRPM4-treated (*siRNA*) cells. Arrowheads indicate currents at 0 mV. **b** Mean current (*I*) - voltage (*V*) relationships for swelling-activated whole-cell currents observed in *Mock* and *siRNA* cells (n = 6–7). Each data point represents the mean ± SEM (vertical bar) of n samples. The mean values at the respective potential for *Mock* and *siRNA* cells did not show a significant difference by statistical analysis. **c** Effects of TRPM4 knockdown on expression of TRPM4 and LRRC8A mRNAs. Left panel shows the PCR products from *Mock* and *siRNA* cells for TRPM4 (top), LRRC8A (middle), and the constitutively transcribed control, GAPDH (bottom). The nucleotide sequences of the PCR products obtained with TRPM4- and LRRC8A-specific primers were completely

identical to the corresponding sequences for TRPM4 (human: NM_017636.4) and LRRC8A (human: NM_001127244), respectively. Right panels show the bar graph representation of the relative expression values of the optical densities in pixels of the PCR bands of TRPM4 (left bars: 41.8 ± 2.9%) and LRRC8A (right bars: 101.2 ± 1.3%) in *siRNA* cells compared to those in *Mock* cells. The values were calculated from five independent PCR amplifications after normalization to the corresponding band of GAPDH control. *P (= 0.0083) < 0.01 compared to *Mock* cell data by t-test.

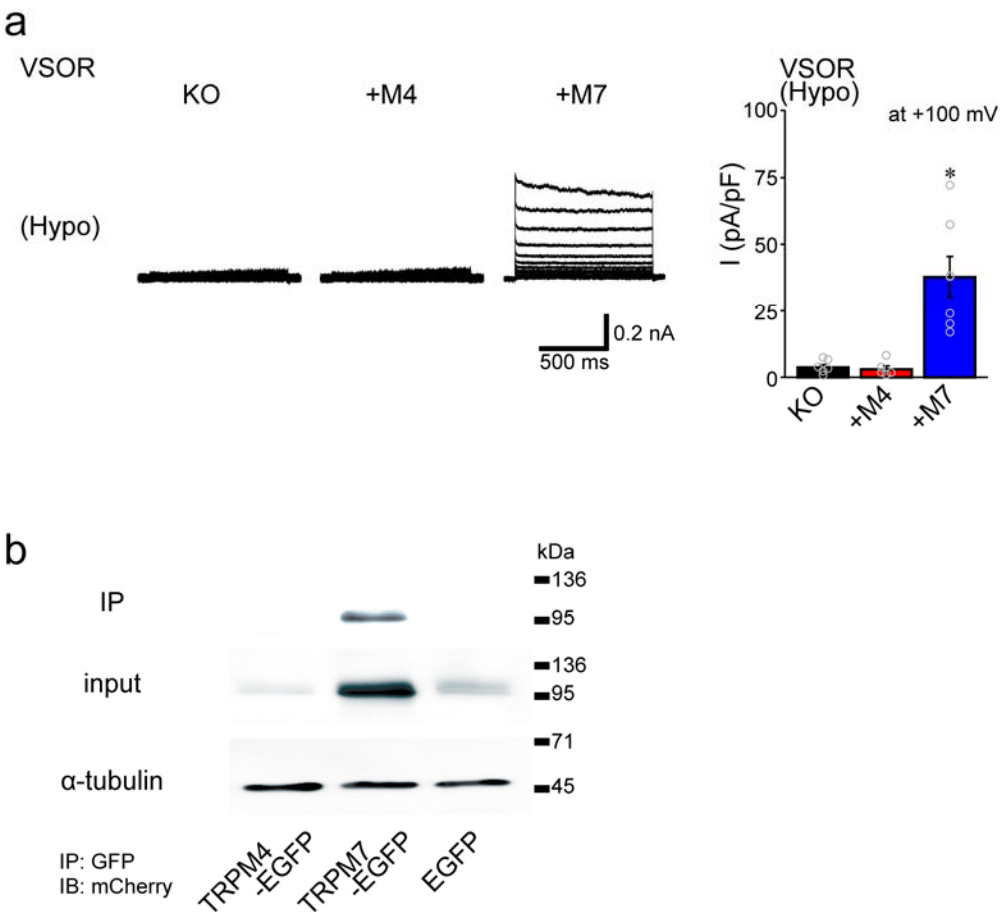

Supplementary Figure 2. Exogenous expression of TRPM4 does not lead to recovery of VSOR currents in TRPM7-deficient DT40 cells and does not exhibit molecular interactions with LRRC8A in HEK293 cells. **a** Representative whole-cell VSOR currents elicited by step pulses in hypotonic conditions (*Hypo*) in gTRPM7-knockout (*KO*) DT40 cells and those transiently expressing with hTRPM4 (*+M4*) and hTRPM7 (*+M7*). (left) The EGFP-positive cells were selected to measure VSOR currents. Peak VSOR current densities recorded at +100 mV (n = 5–7). Each column represents the mean ± SEM (vertical bar) of n samples. **P* (= 0.00082) < 0.001 compared to corresponding *KO* data by one-way ANOVA followed by post-hoc Tukey's test. **b** Co-immunoprecipitation of EGFP, hTRPM4-EGFP, or hTRPM7-EGFP with

LRRC8A-mCherry in transfected HEK293T cells. Immunoprecipitations (*IP*) with an EGFP-specific antibody were subjected to western blot with an antibody to mCherry. For input, aliquots of sample are loaded on a separate gel. The data are representative of two independent experiments.

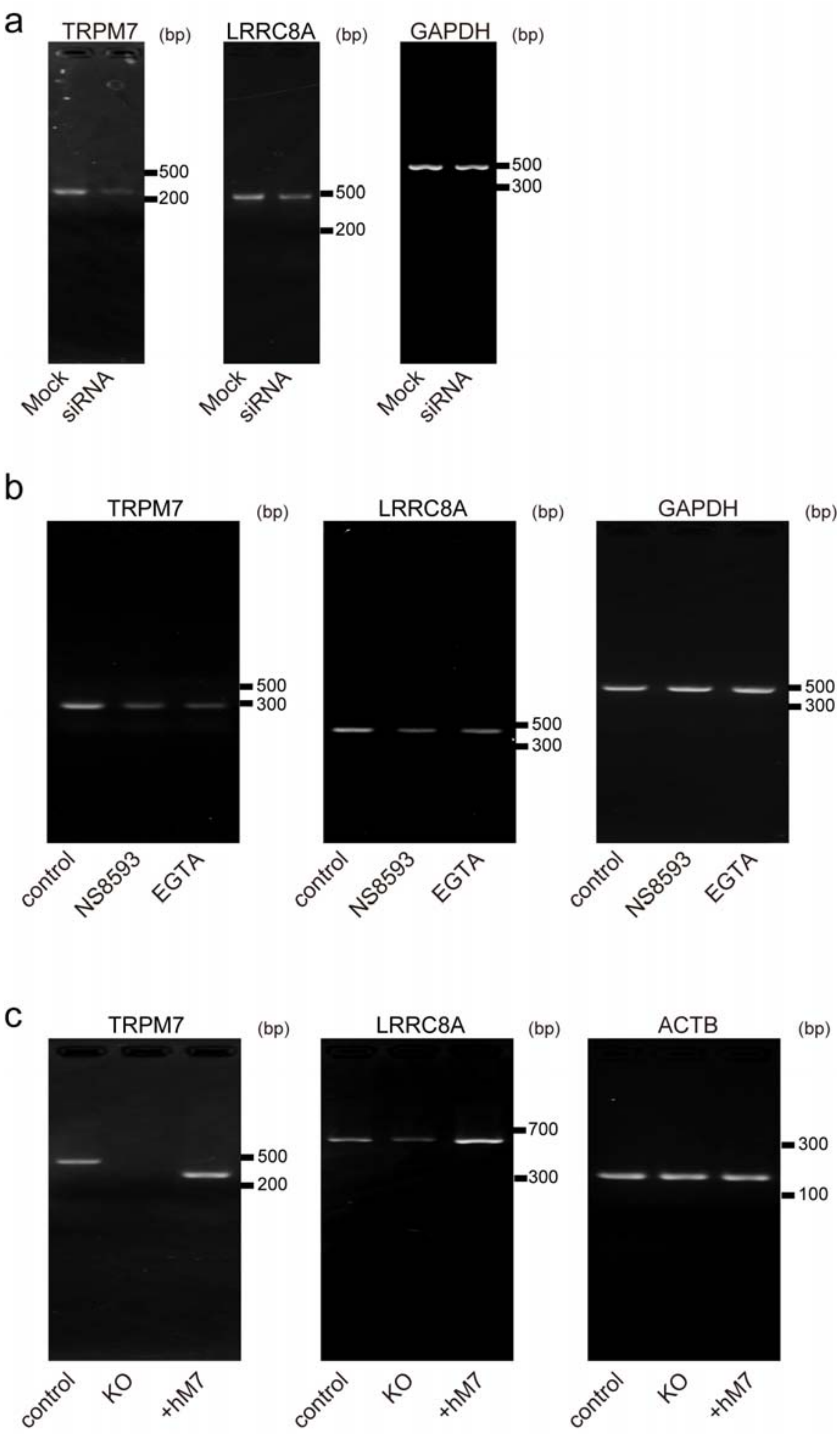

Supplementary Figure 3. Uncropped full-length pictures of agarose gels. a, b and **c** represent the full-length pictures presented in Figs. 1c, 2d, and 7a, respectively.

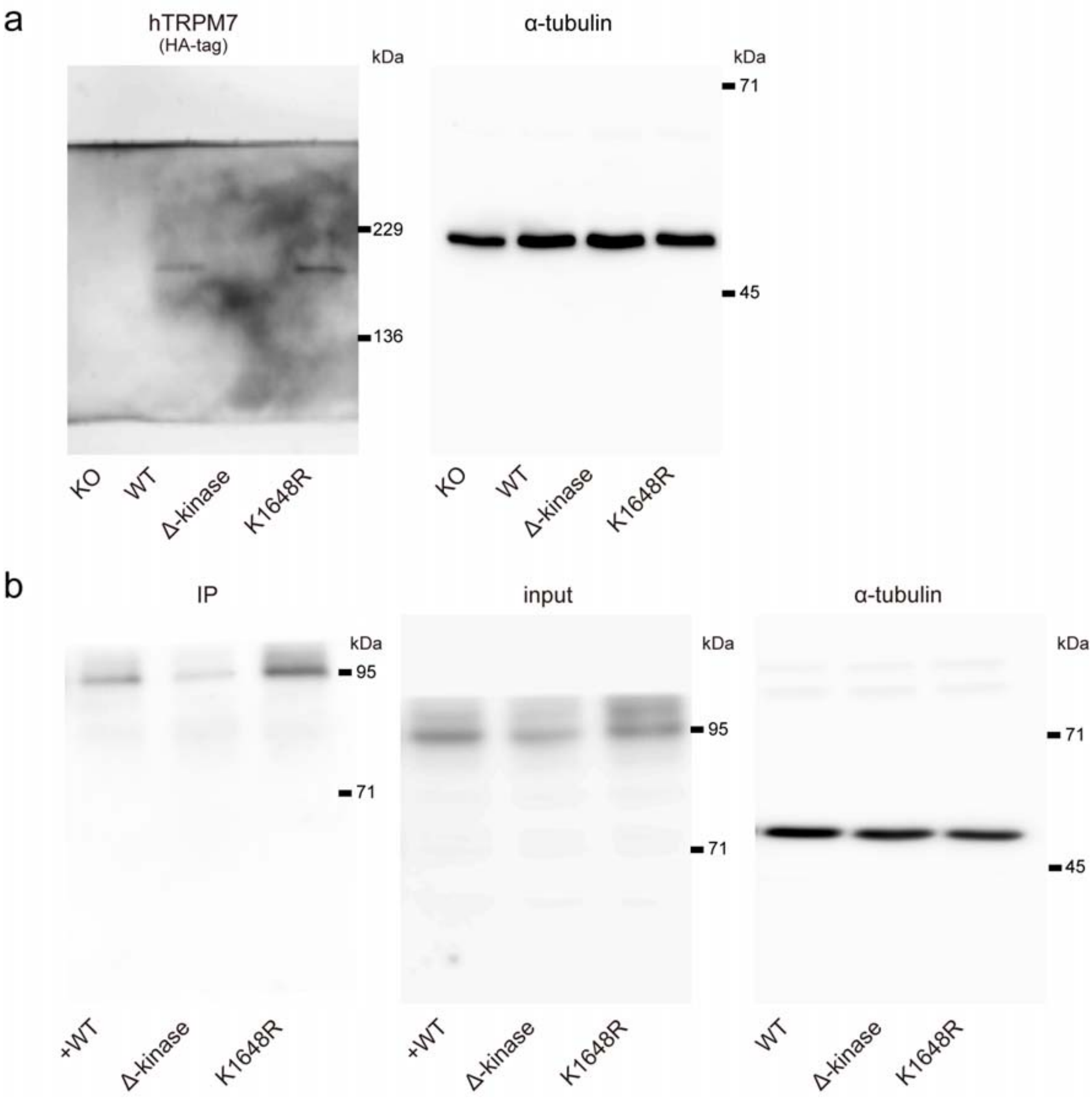

Supplementary Figure 4.Uncropped full-length pictures of western blotting membranes. **a** and **b** represent the full-length pictures presented in Figs. 8c and 9c, respectively.
