# Supplementary material for: TRPM7 is an essential regulator for volume-sensitive outwardly rectifying anion channel
Source: Commun Biol. 2021 May 20;4:599. doi: 10.1038/s42003-021-02127-9 (PMC8137958; doi:10.1038/s42003-021-02127-9)
Supplement: Supplementary file 3 — Description of Supplementary Files [file 42003_2021_2127_MOESM3_ESM.pdf]

Description of Additional Supplementary Files

File name: Supplementary data 1

Description: Numata et al TRPM7-VSOR Supplementary raw data.
