# Supplementary material for: TRPM7 is an essential regulator for volume-sensitive outwardly rectifying anion channel
Source: Commun Biol. 2021 May 20;4:599. doi: 10.1038/s42003-021-02127-9 (PMC8137958; doi:10.1038/s42003-021-02127-9)
Supplement: Supplementary file 5 — Reporting Summary [file 42003_2021_2127_MOESM5_ESM.pdf]

nature research

Corresponding author(s): Yasunobu Okada

Last updated by author(s): Mar 16, 2021

Reporting Summary

Nature Research wishes to improve the reproducibility of the work that we publish. This form provides structure for consistency and transparency in reporting. For further information on Nature Research policies, see our Editorial Policies and the Editorial Policy Checklist.

Statistics

For all statistical analyses, confirm that the following items are present in the figure legend, table legend, main text, or Methods section.

n/a	Confirmed	
☐	☒	The exact sample size (*n*) for each experimental group/condition, given as a discrete number and unit of measurement
☐	☒	A statement on whether measurements were taken from distinct samples or whether the same sample was measured repeatedly
☐	☒	The statistical test(s) used AND whether they are one- or two-sided<br>*Only common tests should be described solely by name; describe more complex techniques in the Methods section.*
☐	☒	A description of all covariates tested
☐	☒	A description of any assumptions or corrections, such as tests of normality and adjustment for multiple comparisons
☐	☒	A full description of the statistical parameters including central tendency (e.g. means) or other basic estimates (e.g. regression coefficient) AND variation (e.g. standard deviation) or associated estimates of uncertainty (e.g. confidence intervals)
☐	☒	For null hypothesis testing, the test statistic (e.g. *F*, *t*, *r*) with confidence intervals, effect sizes, degrees of freedom and *P* value noted<br>*Give P values as exact values whenever suitable.*
☒	☐	For Bayesian analysis, information on the choice of priors and Markov chain Monte Carlo settings
☒	☐	For hierarchical and complex designs, identification of the appropriate level for tests and full reporting of outcomes
☒	☐	Estimates of effect sizes (e.g. Cohen's *d*, Pearson's *r*), indicating how they were calculated

Our web collection on statistics for biologists contains articles on many of the points above.

Software and code

Policy information about availability of computer code

Data collection	pCLAMP10 (Axon), Axopatch 200B amplifier (Axon), CDA-500 (Sysmex), LSM710 (Carl Zeiss), SPARK10M (Tecan Group Ltd), LAS-3000 (Fujifilm),Nanodrop-ND1000 (Thermo Fisher Scientific)
Data analysis	pCLAMP10 (Axon), ZEN (Carl Zeiss), Origin (OriginLab),Image Gauge (Fujifilm)

For manuscripts utilizing custom algorithms or software that are central to the research but not yet described in published literature, software must be made available to editors and reviewers. We strongly encourage code deposition in a community repository (e.g. GitHub). See the Nature Research guidelines for submitting code & software for further information.

Data

Policy information about availability of data

All manuscripts must include a data availability statement. This statement should provide the following information, where applicable:

- Accession codes, unique identifiers, or web links for publicly available datasets
- A list of figures that have associated raw data
- A description of any restrictions on data availability

All the source raw data used for generating main Figures are provided as supplementary files.

Field-specific reporting

Life sciences study design

All studies must disclose on these points even when the disclosure is negative.

Sample size	No sample size calculation was performed. The sample sizes were chosen in order to adequately reflect the variance in the measured effect.
Data exclusions	N/A
Replication	N/A
Randomization	N/A
Blinding	N/A

Reporting for specific materials, systems and methods

We require information from authors about some types of materials, experimental systems and methods used in many studies. Here, indicate whether each material, system or method listed is relevant to your study. If you are not sure if a list item applies to your research, read the appropriate section before selecting a response.

Materials & experimental systems

n/a	Involved in the study	
☐	☒	Antibodies
☐	☒	Eukaryotic cell lines
☒	☐	Palaeontology and archaeology
☒	☐	Animals and other organisms
☒	☐	Human research participants
☒	☐	Clinical data
☒	☐	Dual use research of concern

Methods

n/a	Involved in the study	
☒	☐	ChIP-seq
☒	☐	Flow cytometry
☒	☐	MRI-based neuroimaging

Antibodies

Antibodies used	Anti-HA (11 867 423 001; Roche Diagnostics), anti-GFP (50430-2-AP; Proteintech), anti-mCherry (GTX630189; GeneTex), anti-rat Alexa-488-conjugated (A-11006; Thermo Fisher Scientific), anti-rabbit Alexa-488-conjugated (A-1100; Thermo Fisher Scientific), anti-mouse Alexa-647-conjugated (A-21236; Thermo Fisher Scientific), anti-HA antibody (06340; Nacalai Tesque), anti-α-tubulin (T6074; Sigma-Aldrich)
Validation	Immunohistochemistry: Anti-HA, anti-GFP, anti-mCherry, anti-rat Alexa-488-conjugated, anti-rabbit Alexa-488-conjugated, anti-mouse Alexa-647-conjugated<br>IP and westernblotting: Anti-HA, anti-GFP, anti-mCherry, ECL system (Thermo Fisher Scientific)

Eukaryotic cell lines

Policy information about cell lines

Cell line source(s)	HeLa, HEK293T, DT40, gTRPM7-KO DT40 cells, and hTRPM7-WT-, hTRPM7-K1648R- , hTRPM7-Δ-kinase-gTRPM7-KO DT40 cells.
Authentication	HeLa and HEK293T cell lines was provided from Dr. Okada's lab (NIPS, Japan). DT40 cells and those expressing TRPM7 mutants cell lines were gifted from Dr C. Schmitz and Dr A.-L. Perraud (see: Schmitz C, et al. Cell 114, 191-200 (2003).
Mycoplasma contamination	Mycoplasma contamination was not tested.
Commonly misidentified lines (See ICLAC register)	N/A
